# Metabolic Profile in a Cohort of Young Sicilian Patients with Klinefelter's Syndrome: The Role of Irisin

**DOI:** 10.1155/2022/3780741

**Published:** 2022-04-06

**Authors:** Stefano Radellini, Valentina Guarnotta, Vincenzo Sciabica, Giuseppe Pizzolanti, Carla Giordano

**Affiliations:** Department of Health Promotion, Maternal-Infantile, Internal and Specialist Medicine of Excellence “G. D' Alessandro” (PROMISE), Section of Endocrinology and Metabolism, University of Palermo, Palerm, Italy

## Abstract

Klinefelter's syndrome (KS) is the main cause of hypogonadism and infertility in men and is often related to obesity, metabolic syndrome, and diabetes. The purpose of our real-life observational study was to investigate the metabolic and anthropometric parameters in a population of patients with Klinefelter syndrome compared to a group of healthy age-matched subjects. *Methods*. In our study, 25 consecutive Caucasian adult outpatients (age range 21–52 years, mean age 32.9 ± 12.2) with KS in testosterone replacement therapy and 30 healthy men (age range 25–45 years, mean age 32.4 ± 7.62) were studied. In both groups of subjects, anthropometric indices, lipid profile, glucose metabolic parameters, HbA1c, the homeostasis model assessment estimate of HOMA-insulin resistance (IR), and the insulin sensitivity index (ISI) were evaluated. In addition, we assessed the complete hormonal gonadic status and irisin values in both groups of patients. *Results*. No significant differences were found in BMI and total blood testosterone levels between KS and control subjects. Patients with KS had significantly higher values of WC (*p*=0.028), HbA1c (*p*=0.018), HOMA-IR (*p* < 0.001), FSH (*p* < 0.001), LH (*p* < 0.001), estradiol (*p*=0.001), and irisin (*p*=0.029) and significantly lower HDL-cholesterol (*p*=0.002), AMH (*p* < 0.001), inhibin B (*p* < 0.001), and ISI-Matsuda (*p* < 0.001) compared to healthy controls. Univariate analysis revealed an inverse correlation between irisin and ISI-Matsuda (r = −0.128; *p*=0.010). These data were then confirmed in multivariate analysis. *Conclusions*. KS is characterized by early development of metabolic syndrome and in particular by alterations of the glucose metabolism, independently of testosterone levels serum and BMI. Irisin blood levels of Klinefelter's patients are higher than in healthy subjects and positively correlate with the degree of insulin resistance.

## 1. Introduction

Klinefelter's syndrome (KS) is the most frequent chromosomal pathology of sex chromosomes and represents the main cause of hypogonadism and infertility in men [1]. In addition to disorders of the sexual sphere, this pathology is often related to obesity, metabolic syndrome, and diabetes mellitus [2, 3]. Although it has been widely demonstrated that hypotestosteronemia is an independent risk factor of development of central obesity and insulin resistance [4, 5], in KS, testosterone replacement therapy (TRT) is not capable of improving these metabolic aspects as effectively as for other forms of hypogonadism [6, 7], suggesting a more complex etiology still not fully clarified [2, 4, 8]. The adipose tissue is abundantly present in the bodies of patients with KS [2], especially as visceral fat, and the role of adipose tissue as an endocrine organ able to determine the metabolic risk of these patients needs to be clarified. Irisin is a myokine involved in the thermogenesis of adipose tissue, capable of inducing the conversion of white adipose tissue (WAT) into brown adipose tissue (BAT) [9]. Various studies have investigated a possible etiopathogenetic role of irisin in type 2 diabetes and metabolic syndrome, though with discordant results [10–12]. However, few studies have investigated the metabolic effects of irisin in patients with hypogonadism and specifically with KS [13, 14]. The purpose of the present real-life observational study was to investigate the metabolic, anthropometric parameters, and irisin levels in a population of patients with Klinefelter's syndrome compared to a group of healthy subjects.

## 2. Materials and Methods

In the period from September 2019 to September 2020, 25 consecutive Caucasian adult outpatients (age range 21–52 years, mean age 32.9 ± 12.2) with KS and 30 healthy men (age range 25–45 years, mean age 32.4 ± 7.62) were recruited. Of the 25 patients with KS, 23 had the classical karyotype 47XXY, 1 the karyotype 48XXYY, and another the karyotype 46XX, t (Y), p (15). All patients with KS had serum total testosterone values in the normal reference range for age during TRT with transdermal testosterone gel (40–60 mg daily) in the six months prior to enrollment. Patients affected by severe obesity and type 2 diabetes or treated with insulin-sensitizing drugs for insulin resistance were excluded. All patients who practiced regular physical activity or performed intense aerobic physical activity (over 90 minutes/week) were excluded. To assess adherence to this instruction, capable of interfering with irisin values, a pedometer was provided to each patient for 7 days, so that no more than 5000 steps per day were practiced. The study was approved by a local ethics committee and conducted with the informed consent of all patients, in agreement with the Helsinki Declaration.

### 2.1. Study Design

Both groups of subjects (control group and KS group) underwent the following endocrine metabolic investigations:

- Blood sampling was performed in the morning using Greiner Bio-One Vacuette Z tubes, and 9 mL of blood was withdrown, with at least 8 hours of postabsorptive fasting, for evaluation of total testosterone (TT), 17beta-estradiol (E2), follicular stimulating hormone (FSH), luteinizing hormone (LH), prolactin, inhibin B, anti-Müllerian hormone (AMH), and irisin. For patients on hormone replacement therapy, the sample was obtained after 2 hours of transdermal administration.
- Testicular volumetry by the Prader orchidometer.
- Measurement of anthropometric indices, weight, height, body mass index (BMI), and waist circumference (WC).
- Blood sampling was performed in the morning, with at least 8 hours of postabsorptive fasting, for evaluation of glucose and insulin levels at times 0', 30', 60', 90', and120' during the oral glucose tolerance test (OGTT), glycosylated hemoglobin (HbA1c), total cholesterol, high-density lipoprotein cholesterol (HDL), and triglycerides.

As a surrogate index of insulin sensitivity, we performed the homeostasis model assessment estimate of insulin resistance (HOMA-IR) [15], the insulin sensitivity index (ISI), an indirect index derived from the OGTT and validated by Matsuda and DeFronzo [16], and the oral disposition index (DIo), an indicator of *β*-cell function relative to insulin sensitivity [17].

### 2.2. Laboratory Methods

Glucose and lipids were measured by standard colorimetric methods (Modular P8000, Roche, Milan, Italy). Low-density lipoprotein cholesterol (LDL-C) levels were calculated with the Friedewald formula [18]. Hormone measurement was performed with standard immunometric methods using enhanced electrochemiluminescence (Modular P8000, Roche, Milan, Italy).

### 2.3. Irisin Dosage Method

Irisin was quantized using a commercial enzyme immunoassay kit (EK-067-29; Phoenix Pharmaceuticals, Inc., Karlsruhe, Germany), as per protocol. The lowest detectable irisin concentration was 1.77 ng/ml and the highest was 1000 ng/ml (dynamic range is from 0.1 to 1000 ng/ml). The kit used in the study was validated against Western blotting and mass spectrometry.

### 2.4. Calculated Indexes Methods

HOMA-IR was calculated as fasting insulin (micro U/l) *x* fasting glucose (nmol/l)/22.5. The Matsuda index of insulin sensitivity (ISI-Matsuda) was calculated as (10000/glucose (mg/dl) *x* insulin (mU/ml) *x* glucose mean *x* insulin mean) [16], and the oral disposition index (DIo) was calculated as ((Δinsulin0–30/Δglucose0–30) *x* (1/fasting insulin)) [17].

### 2.5. Statistical Analysis

The data analysis was conducted using the Statistical Packages for Social Science SPSS version 19 (SPSS, Inc., IBM, Italy). The normality of the quantitative variables was tested with the Shapiro–Wilk test. The initial characteristics of the two groups are presented as mean value ± standard deviation for continuous variables, while percentages and proportions were used for categorical variables. The differences between the two independent groups were evaluated with Student's *t*-test. Univariate correlations were evaluated by Pearson's test. Multiple linear regression was performed to identify predictors of irisin. The variables that were statistically significant in the univariate analysis were included in the linear multiple regression model. *P* value <0.05 was considered statistically significant.

## 3. Results

**Figure 1:**
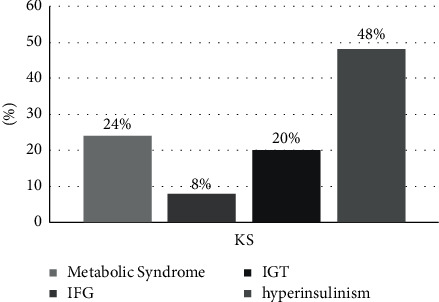
Prevalence of metabolic alterations in the subjects with KS examined. IFG, impaired fasting glucose; IGT, impaired glucose tolerance.

Metabolic syndrome was found in 24% of patients with KS, in accordance with the criteria of the ATP III-NCEP (Adult Treatment Panel III-National Cholesterol Education Program) association. In the group of patients with KS, alterations of the glucose metabolism such as impaired fasting glucose (IFG) and impaired glucose tolerance (IGT) were found, respectively, in 8% and 20% of the total patients examined (Figure 1). 52% of Klinefelter's patients were normal weight, 16% overweight, and 32% had first-class obesity. The anthropometric, metabolic, and hormonal characteristics of the two age-matched patient groups are given in Table 1. No significant differences were found for BMI and total blood testosterone levels. Patients with KS had significantly higher values of WC (*p*=0.028), HbA1c (*p*=0.018), HOMA-IR (*p* < 0.001), FSH (*p* < 0.001), LH (*p* < 0.001), estradiol (*p*=0.001), and irisin (*p*=0.029) and significantly lower HDL-cholesterol (*p*=0.002), AMH (*p* < 0.001), inhibin B (*p* < 0.001), ISI-Matsuda (*p* < 0.001), and DIo (<0.01) compared to healthy controls.

**Figure 2:**
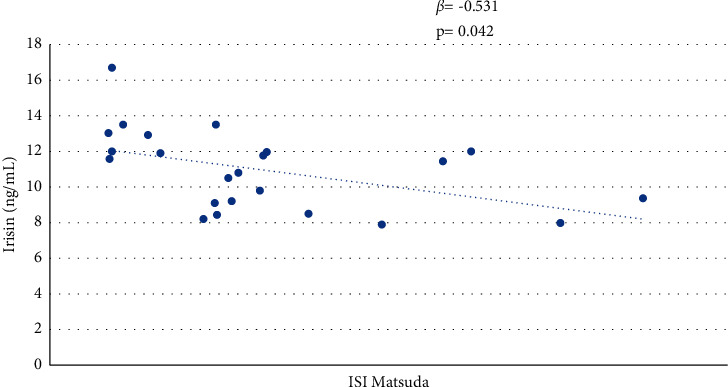
Correlation between irisin and ISI-Matsuda in patients with Klinefelter syndrome.

In the group of patients with KS, no significant difference was found in ISI-Matsuda between the normal weight and the overweight-obese patients. The univariate analysis revealed an inverse correlation between irisin and ISI-Matsuda (*r* = −0.128; *p*=0.010) (Table 2). This datum was then confirmed in the multivariate analysis (Figure 2).

## 4. Discussion

Evaluation of the anthropometric parameters of the Klinefelter patient group confirmed that this syndrome determines a metabolically unfavorable body composition, with elevations of visceral adiposity, regardless of hypogonadism. Although these patients had mean BMI values comparable to the control group, the waist circumference values were significantly higher. In our study, the patients with KS were young and had normal blood testosterone levels during stable TRT, matched with testosterone levels of the healthy control group, but had a significantly worse metabolic profile, in terms of low HDL-cholesterol levels, lower ISI-Matsuda, lower DIo, and higher HOMA-IR, confirming a specific negative metabolic impact of KS per se. Bojesen et al. showed that visceral obesity represents the main risk factor for the development of insulin resistance and metabolic syndrome in patients with KS, regardless of testosterone levels [19]. Following the scientific literature, in our study, this was confirmed by increased insulin resistance in KS patients, as demonstrated by significantly higher HOMA-IR and lower ISI-Matsuda values compared to healthy controls. From an analysis of the Klinefelter subgroups, no differences emerged, in terms of insulin resistance, between the normal weight group and the overweight-obese group, indicating major alterations of the glucose metabolism typical of KS, which is maintained in the various classes of BMI, with mechanisms still to be clarified. In agreement with these findings, in a recent study, Hans et al. documented the occurrence of glucose metabolism alterations even for lower BMI values in patients with KS [20]. Pasquali et al. evaluated the effects of hormone treatment replacement in 48 KS patients with metabolic syndrome. After 3 years of treatment, no changes in the metabolic profile were ascertained [8]. In agreement to these data, we documented an altered metabolic profile in patients with KS, although well treated with TRT compared to healthy control subjects. In our study, serum irisin levels were higher in the patient population with KS than in healthy subjects. To date, no data regarding the levels of this myokine in patients with karyotype 47XXY are available in the scientific literature. In recent years, numerous studies have been carried out on irisin, many of which have suggested a possible protective effect of this myokine in the development of the metabolic syndrome and diabetes mellitus; however, the results are often contradictory. Most of the published studies correlate the metabolic syndrome and diabetes mellitus with low irisin levels [21, 22].

However, in this regard, the data present in the literature are conflicting. Some studies have shown an increase in serum levels of irisin in subjects with DM2 [10] and their inverse relationship with insulin sensitivity [11]. Crujeiras et al. found a direct relationship between irisin and BMI, waist circumference, and fat mass, assuming a compensatory increase in irisin to counteract insulin resistance [23]. These data also seem to be confirmed in our group of patients with KS, where a significantly negative correlation between irisin levels and insulin sensitivity expressed in terms of ISI-Matsuda was demonstrated. In our study, we found that serum irisin levels were significantly higher in the patient with KS than in healthy subjects. This result could be explained in various ways. Several authors investigated the difference in irisin levels in the two genders and found significantly higher levels in females [24–26]. This was also confirmed by Fukushima et al. and was attributed to a likely stimulatory effect of estradiol on the muscle, as well as the different distribution of body fat that characterizes the two sexes [27]. In our study, patients with KS had statistically higher levels of estradiol than controls, but the bivariate analysis did not show a significant correlation between irisin and estradiol or between irisin and testosterone. In accordance with another study documented significantly higher levels of irisin in hypogonadal men [13], our data demonstrated higher levels of irisin in patients with KS than in control subjects.

## 5. Conclusions

Klinefelter's syndrome predisposes people to the early development of the metabolic syndrome and in particular to alterations of the glucose metabolism, independently of the testosterone levels and BMI. From our data, we can hypothesize that irisin levels are correlated with hypogonadism. Future studies on a larger number of patients could confirm this hypothesis.

## Figures and Tables

**Table 1:** Comparison of anthropometric, metabolic, and hormonal parameters between patients with Klinefelter's syndrome and healthy controls.

	Controls (*N* = 30)	Patients with KS (*N* = 25)	*P*
	Mean ± SD	Mean ± SD	
Age (years)	32.4 ± 7.62	32.9 ± 12.2	0.886
BMI (Kg/m^2^)	26.26 ± 3.41	27.35 ± 7.02	0.515
WC (cm)	92.78 ± 9.68	102,8 ± 18.41	0.028
Total cholesterol (mmol/l)	4.95 ± 0.78	4.51 ± 0.95	0.064
HDL-cholesterol (mmol/l)	1.48 ± 0.17	1.22 ± 0.31	0.002
Triglycerides (mmol/l)	1.21 ± 0.32	1.30 ± 0.87	0.599
LDL-cholesterol (mmol/l)	2.91 ± 0.79	2.69 ± 0.77	0.303
GOT (U/l)	18.4 ± 6.5	19 ± 7.74	0.716
GPT (U/l)	22.3 ± 8.43	23.05 ± 13.22	0.799
HbA1c (%)	5.2 ± 0.38	5.48 ± 0.44	0.018
Fasting glucose (mmol/l)	4.07 ± 0.20	4.7 ± 0.79	0.388
Fasting insulin (mUI/l)	9.42 ± 5.64	11.83 ± 6.52	0.151
HOMA index IR	2.2 ± 1.5	7.81 ± 5.57	<0.01
ISI-Matsuda	5.9 ± 3.8	1.87 ± 1.29	<0.01
Oral disposition index	2.41 ± 1.32	−0.75 ± 4.74	<0.01
Total testosterone (ng/dl)	551.41 ± 287.2	465.12 ± 319.31	0.296
FSH (mUI/ml)	5.3 ± 2.9	27.75 ± 14.26	<0.001
LH (mUI/ml)	4.01 ± 2.3	17.36 ± 9.71	<0.001
AMH (ug/l)	8.5 ± 3.4	0.09 ± 0.05	<0.001
Inhibin B (ng/l)	128.4 ± 54.7	<25 ± 0.01	<0.001
PRL (ug/l)	9.4 ± 5.1	12.6 ± 8.08	0.784
E2 (ng/l)	22.3 ± 3.5	30.21 ± 12.23	0.001
Irisin (ng/ml)	10.28 ± 1.69	11.79 ± 1.95	0.029

WC, waist circumference; BMI, body mass index; HDL, high-density lipoprotein; LDL, low-density lipoprotein; GOT, glutamic oxaloacetic transaminase; GPT, glutamate pyruvate transaminase' FSH, follicular stimulating hormone; LH, luteinizing hormone; PRL, prolactin; AMH, anti-Müllerian hormone; E2, 17beta-estrasiol.

**Table 2:** Correlation between Irisin and clinical and hormonal metabolic parameters (univariate analysis) in patients with Klinefelter syndrome.

Irisin	
Klinefelter syndrome	*r*
WC (cm)	0.377
BMI (Kg/m^2^)	0.269
ISI-Matsuda	−0.128
HbA1c (%)	−0.041
FSH (mUI/ml)	0.005
LH (mUI/ml)	0.152
E2 (pg/ml)	0.147
Total testosterone (ng/ml)	−0.014

WC, waist circumference; BM, body mass index; FSH, follicular stimulating hormone; LH, luteinizing hormone; E2, 17beta-estradiol.

## Data Availability

The data used to support the findings of this study are available from the corresponding author upon request.
